# The geographical distribution patterns of *Chrysoteuchia* Hübner in China and description of a new species (Lepidoptera, Crambidae)

**DOI:** 10.3897/zookeys.853.34149

**Published:** 2019-06-06

**Authors:** Lu-Lan Jie, Jing-Bo Yang, Wei-Chun Li

**Affiliations:** 1 College of Agronomy, Jiangxi Agricultural University, Nanchang, 330045, China Jiangxi Agricultural University Nanchang China

**Keywords:** ArcGIS, Crambinae, MaxEnt, Pyraloidea, taxonomy

## Abstract

The geographical distribution patterns of *Chrysoteuchia* Hübner in China are analysed with MaxEnt and ArcGIS based on known localities and nineteen environmental variables. The results suggest that southeastern China is a highly suitable area, and Bio11 (mean temperature of the coldest quarter), Bio12 (annual precipitation) and Bio18 (precipitation of the warmest quarter) are revealed to be the main variables affecting the present distribution patterns. Among them, Bio18 is the strongest predictor with a 24.3% contribution. Furthermore, a new species from Tibet is added to the genus, *Chrysoteuchialandryi***sp. nov.**, and the male of *C.curvicavus* is described for the first time. Images of adults and their genitalia are illustrated, and two maps showing the geographical distribution patterns of *Chrysoteuchia* in China are provided.

## Introduction

*Chrysoteuchia* was erected by Hübner (1825) with *Tineahortuella* Hübner, 1796 as the type species. Morphologically, *Chrysoteuchia* species are variable in wing pattern, but can be recognised with characters of the genitalia: in males, the well-developed sacculus is adorned with a projection while in females the papillae anales have a concave posterior margin, the posterior apophyses are slender, and the anterior apophyses are absent (Li and Li 2010).

The genus has 35 species with Palearctic, Sino-Japanese, and Oriental distributions except for *C.topiaria* (Zeller, 1866), which is endemic to the Nearctic region (Bleszynski 1965; Chen et al. 2001, 2003; Inoue 1989; Landry 1995; Li and Li 2010; Li and Liu 2012). In China, the genus has an exceptional diversity with 33 species (Li and Li 2010; Li and Liu 2012). Prior to this study, most known localities of Chinese *Chrysoteuchia* were reported to occur in eastern China (Li and Li 2010), but this geographical pattern was never analysed. In the analysis of geographical patterns, MaxEnt (Phillips et al. 2006) has been used previously as an effective model for predicting the potential distribution of various taxa (Li 2017, 2018, 2019). In the present paper, we employ MaxEnt and ArcGIS to analyse the distribution of *Chrysoteuchia* in China. We also describe a new species from Galongla Snow Mountain, Tibet.

## Materials and methods

All specimens were collected at night with a mercury-vapour lamp. The specimens were hand-collected alive and killed with vapours of ammonium hydroxide prior to mounting and spreading as shown in Landry and Landry (1994). The morphological terminology follows Landry (1995). Illustrations of adults and genitalia were prepared with a digital camera attached to a Zeiss SteREO Discovery V12 microscope and to an Optec BK-DM320 microscope, respectively.

The potential geographic distribution of *Chrysoteuchia* was predicted using MaxEnt (Phillips et al. 2006) based on known localities from the literature (Bleszynski 1965; Chen et al. 2001, 2003; Li and Li 2010; Li and Liu 2012) and the collection localities of the specimens examined in this study (see Suppl. material 1: Table S1); nineteen environmental variables (Table 1) were retrieved from the WorldClim database (http://www.worldclim.org) at a resolution of 2.5 arc-min (Hijmans et al. 2004). MaxEnt was set with 10,000 as the maximum number of background points and 75% training data. The relative importance of each variable was evaluated by contribution in percentage. The cartographic illustrations were created using ArcGIS 10.1. The logistic values of potential habitats were set to 0–1.

**Table 1.** Environmental variables used in the study and their contribution in percentage and permutation importance.

Code	Environmental variables	Unit	Contribution in percentage	Permutation importance
Bio1	Annual mean temperature	°C	0.1	0.1
Bio2	Mean diurnal range (mean of monthly max. and min. temperatures)	°C	9.9	0.2
Bio3	Isothermality ((Bio2/Bio7) × 100)	–	8.2	6.2
Bio4	Temperature seasonality (standard deviation ×100)	C of V	4.6	4.2
Bio5	Maximum temperature of the warmest month	°C	2	6.7
Bio6	Minimum temperature of the coldest month	°C	0.7	4.2
Bio7	Temperature annual range (Bio5–Bio6)	°C	0	0.5
Bio8	Mean temperature of the wettest quarter	°C	2.9	5
Bio9	Mean temperature of the driest quarter	°C	0.1	7
Bio10	Mean temperature of the warmest quarter	°C	0.8	0
Bio11	Mean temperature of the coldest quarter	°C	**16.5**	12.6
Bio12	Annual precipitation	mm	**21.3**	1.7
Bio13	Precipitation of the wettest period	mm	0	0.6
Bio14	Precipitation of the driest period	mm	0.3	3.6
Bio15	Precipitation seasonality (CV)	C of V	2.6	6.9
Bio16	Precipitation of the wettest quarter	mm	4	3.1
Bio17	Precipitation of the driest quarter	mm	1.5	5.8
Bio18	Precipitation of the warmest quarter	mm	**24.3**	**31.4**
Bio19	Precipitation of the coldest quarter	mm	0	0

## Results

### Geographical patterns of distribution of *Chrysoteuchia*

**Figure 1.**
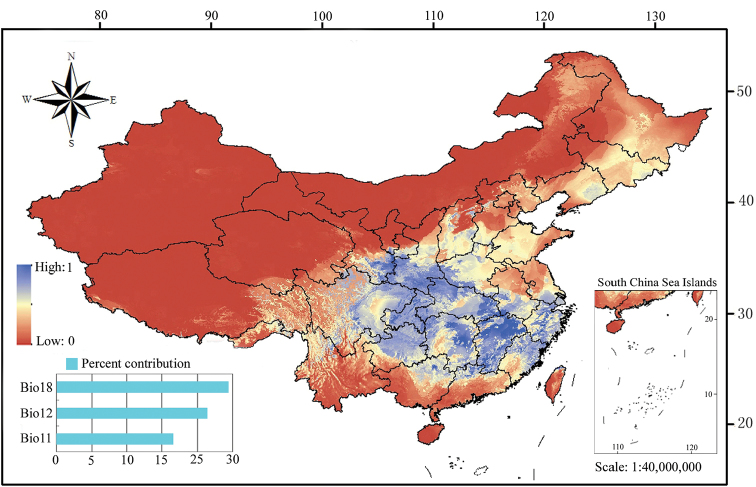
Potential distribution of *Chrysoteuchia* in China. Histograms show the contribution in percentage of the important variables affecting the distribution patterns. The rainbow bar indicates logistic values of potential habitats.

The geographical patterns of distribution of Chinese *Chrysoteuchia* were analysed with MaxEnt based on all the known localities in China (Suppl. material 1: Table S1) and nineteen environmental variables (Table 1). Based on the results illustrated with ArcGIS (Fig. 1), we can recognise the mediocre and more suitable regions for *Chrysoteuchia* species, located in humid to semi-humid areas, generally called the monsoon regions in eastern China. Among the environmental variables, our statistics show that Bio11 (mean temperature of the coldest quarter), Bio12 (annual precipitation), and Bio18 (precipitation of the warmest quarter) are the main variables affecting the geographical distribution of the genus (Fig. 1). Among them, Bio18 is revealed to be the strongest predictor with a 24.3% contribution (Table 1).

**Figure 2.**
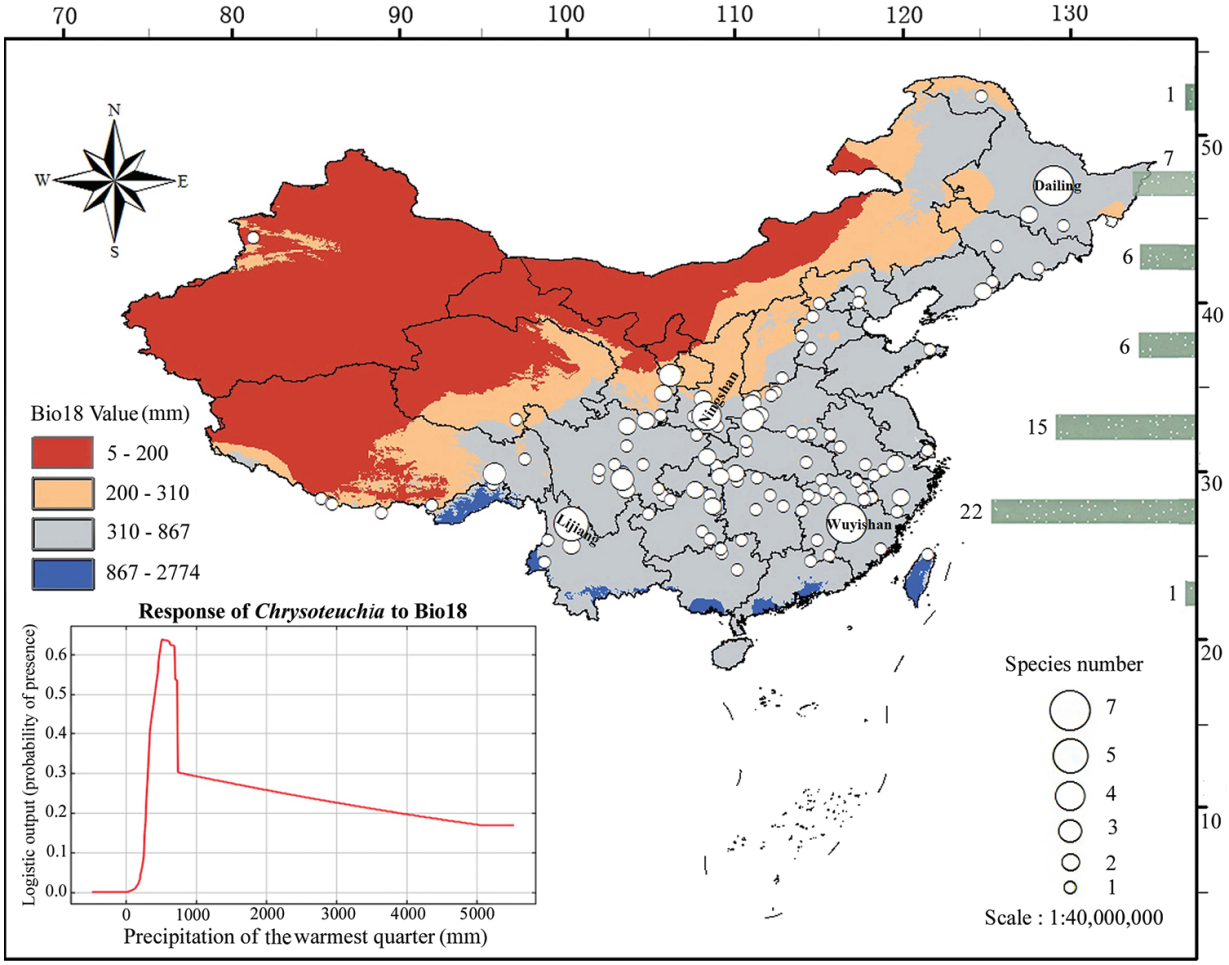
Geographical distribution of *Chrysoteuchia* in China and precipitation of the warmest quarter (Bio18). White circles indicate surveyed sites and number of species per site. Green bars show the known numbers of species at every 5° between 20°N and 55°N.

We illustrate all the known collecting localities of the genus in China by mapping the strongest predictor, i.e. Bio18 (Fig. 2). Dailing in Heilongjiang Province (129°02'E, 47°02'N) and Wuyishan in Fujian Province (116°42'E, 26°54'N) have the highest numbers of species (7 species) (Fig. 2). The second and third highest species diversity at a single locality were found at Lijiang in Yunnan Province (100°14'E, 26°52'N) and Ningshan in Shaanxi Province (108°20'E, 33°19'N), with 5 and 4 species respectively (Fig. 2). To further clarify the distribution patterns of the genus in China, we plotted the known numbers of species at every 5° between 20°N and 55°N (Fig. 2). The detailed results for each region are as follows: 20°N–25°N (1 species), 25°N–30°N (22 species), 30°N–35°N (15 species), 35°N–40°N and 40°N–45°N (6 species each), 45°N–50°N (7 species), and 50°N–55°N (1 species). In suitable areas, the general tendency in species richness of the genus decreases as the latitude increases. In addition, the suitable habitats and almost all known localities of the genus are located in the regions with 310–867 mm precipitation of the warmest quarter, which is supported by the response curve of *Chrysoteuchia* to Bio18 (Fig. 2). Thus, there is a high correlation between the distribution patterns of the genus and Bio18.

### Taxonomic account

#### Chrysoteuchia landryi sp. nov.

Taxon classificationAnimaliaLepidopteraCrambidae

http://zoobank.org/F7167000-855D-4BF2-8333-683B8123CC93

Figs 3–7

##### Type material.

***Holotype*** ♂: CHINA: the foot of Galongla Snow Mountain (29°44.29'N, 95°40.61'E), Mêdog, Tibet, 3415 m, 22.vii.2014, Wei-Chun Li leg., genital prep. no. LW15049 (JXAUM).

***Paratypes***: 2 ♂♂, 2 ♀♀, same data as the holotype, genital prep. nos. LW15007, LW15059 (JXAUM).

##### Differential diagnosis.

This new species is similar to *Chrysoteuchiapicturatella* (South, 1901), *C.gonoxes* (Bleszynski, 1962), and *C.dentatella* Song & Chen, 2001 in having an apical prong on the sacculus and a well-developed apical spine on the phallus in the male genitalia. In female genitalia, it also resembles the above three species in having two lateral spines on the posterior margins of the lamella postvaginalis, and double signa on the corpus bursae. However, the new species can be easily distinguished by lacking fasciae on the forewing (Fig. 5), the presence of a crescent-shaped protuberance on the costa of the valva in male genitalia (Fig. 6), and the female antrum ending with two small triangular projections on the lateral margins (Fig. 7). In the latter three species, the forewing fasciae are well developed, the costa of the valva is armed with spine-like projections, and the antrum is without distal spines (Bleszynski 1965; Song and Chen 2001).

**Figures 3–7.**
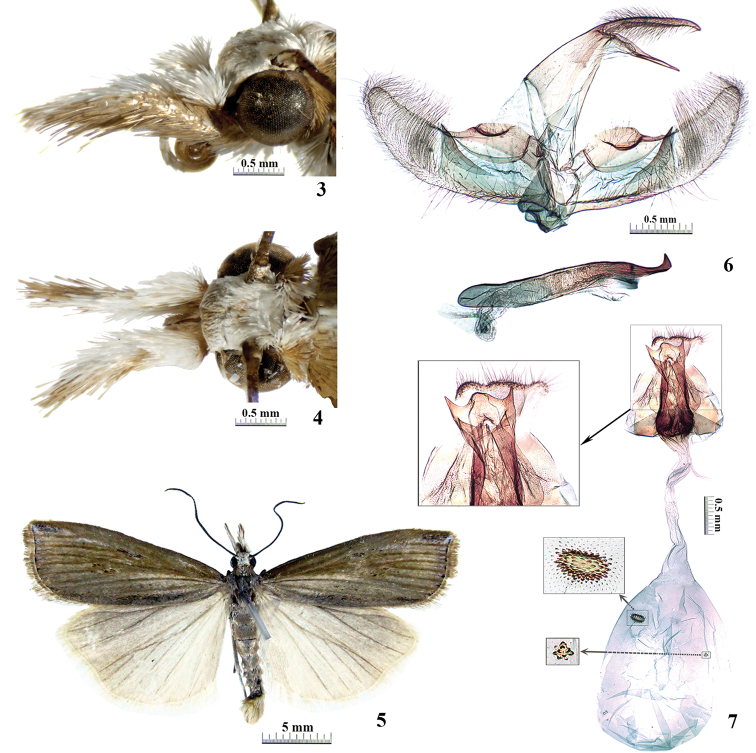
*Chrysoteuchialandryi* sp. nov. **3–6** holotype, male **7** paratype, female **3** head in lateral view **4** head in dorsal view **5** adult **6** male genitalia **7** female genitalia.

##### Description.

***Adult*** (Figs 3–5): Forewing length 11.0–13.0 mm. Frons white mixed with pale brown. Vertex white. Labial palpus approximately twice as long as compound eye diameter, pale brown on outer side, white on dorsal and inner sides. Maxillary palpus white, basally pale brown. Antenna scapus white mixed with pale brown; flagellomere blackish brown. Patagium and tegula pale brown. Thorax blackish brown. Forewing densely covered with brown scales, apex suffused with black and white scales; termen of apex black, four terminal black dots running from middle of termen to tornus; cilia pale brown. Hindwing greyish white, suffused with pale brown scales around apex and along veins; cilia greyish white.

***Male genitalia*** (Fig. 6): Uncus thin and long, tapering to blunt apex, tip slightly curved downward on lateral view. Gnathos straight, a bit shorter than uncus, tapering to point tip. Tegumen approximately twice as long as gnathos, with broad dorsal bridge. Valva broad at basal half, distal half narrowing towards apex, apex rounded. Costa with crescent-shaped protuberance near base, basal half strongly sclerotised and gently convex, concave near middle. Sacculus basally narrow, broadened towards distal prong; distal prong nearly triangular, tip pointed and reaching costa. Juxta ovate. Saccus broad, concave at middle of distal margin. Phallus slightly shorter than valva, apical spine well-developed, ending with triangular prong; cornutus absent.

***Female genitalia*** (Fig. 7): Papillae anales broad, concave on posterior margin. Tergite VIII coalescing with antrum. Lamella postvaginalis developed, slightly broader than antrum, medially convex, posterolaterally with long spine. Antrum strongly sclerotised, approximately three times as thick as median part of ductus bursae, ending with two small triangular projections at lateral sides. Ductus bursae long and thin, membranous; ductus seminalis arising from posterior one fourth of ductus bursae. Corpus bursae ovate; signa double, oblong and lotus flower-shaped, consisted of tiny spines with various sizes.

##### Distribution.

Currently only found at Galongla Snow Mountain, in Mêdog County, Tibet of China.

##### Natural history.

Unknown except that the moths are in flight in late July and come at light. The habitat of this species is identical to that of *Metaeuchromiusglacialis* Li, 2015 and *Scoparia* spp., collected at the foot of Galongla Snow Mountain. Most parts of the mountain are covered with snow; the vegetation at the bottom is a blend of alpine meadows, shrubs, and conifers on the south slope (Li and Liu 2015; Li et al. 2016).

##### Etymology.

In honour of Dr Bernard Landry, who contributed profoundly to systematic research on the subfamily Crambinae, and who substantially contributes to the catalogue of the world Crambinae species in GlobIZ (www.pyraloidea.org).

#### Chrysoteuchia curvicavus

Taxon classificationAnimaliaLepidopteraCrambidae

Song & Chen, 2001

Figs 8–10

Chrysoteuchia curvicavus Song & Chen in Chen et al. 2001: 186, figs 5, 11. Type locality: Wuyishan, Fujian Province, China. Type depository: Institute of Zoology, Chinese Academy of Sciences, Beijing.

##### Specimens examined.

14 ♂♂, 25 ♀♀: CHINA: Dafengding Nature Reserves, Mabian (28°51'N, 103°31'E), Sichuan Province, 1100 m, 9–10.viii.2014, Wei-Chun Li leg., genital prep. no. WD17022 (JXAUM).

##### Description.

**Figures 8–10.**
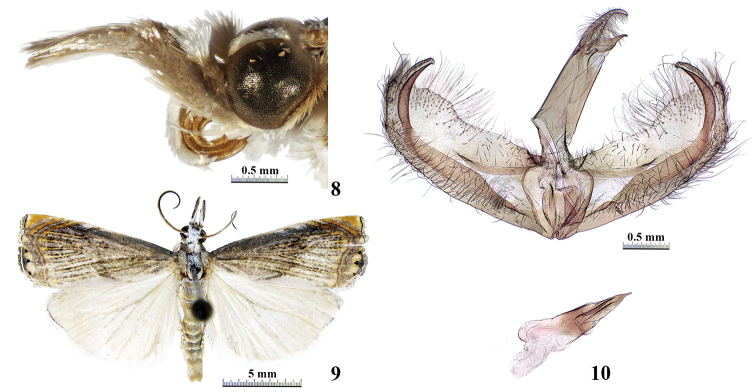
*Chrysoteuchiacurvicavus* Song & Chen **8** head in lateral view, male **9** adult, male **10** male genitalia.

***Male adult*** (Figs 8, 9): Forewing length 9.5–11.0 mm. Frons and vertex white. Maxillary palpus pale brown, ending with white. Labial palpus pale brown. Antenna scapus white mixed with grey dorsally, pale brown ventrally; flagellomere pale brown and white alternately on dorsal surface, pale brown on ventral surface. Forewing ground colour white, costa densely covered with blackish brown scales between base and subterminal fasciae, the remaining suffused with sparse blackish brown scales along veins; median fascia blackish brown, angled outwards at anterior one fourth; two subterminal fasciae yellowish brown, out-curved at anterior one third; terminal area pale yellow; terminal fascia black, with three evenly spaced black spots; cilia shiny, pale brown, with greyish white basal line. Hindwing and cilia greyish white. Abdomen pale brown.

***Male genitalia*** (Fig. 10): Uncus apically curved downwards in lateral view, tapering to blunt apex. Gnathos basally broad, tapering to point tip, a bit shorter than uncus. Tegumen nearly four times as long as gnathos, with narrow dorsal bridge. Valva with sclerotised basal line near middle, apical quarter nearly triangular. Costa concave at approximately basal three-fifths. Sacculus narrow and thin, distal prong well-developed and reaching beyond apex of valva. Juxta heart-shaped, basely narrow, broadened towards tip, distal margin slightly concave. Phallus approximately half as long as valva, basal half broad, distal half narrowing towards tip and armed with two sclerotised wrinkles, apex pointed; cornutus absent.

##### Distribution.

China (Sichuan, Fujian).

##### Remarks.

The male of *C.curvicavus* is described for the first time. This species is similar to *C.atrosignata* (Zeller, 1877) in having an apical prong on the sacculus and a pointed apex on the phallus in the male genitalia. However, it can be easily distinguished by the distal prong of the sacculus reaching beyond the apex of the valva, and the phallus approximately half as long as the valva and armed with two sclerotised wrinkles (Fig. 10). In the latter species, the distal prong of the sacculus reaches beyond the costa at the basal three-fifths of the valva and the phallus is nearly as long as the valva and without sclerotised wrinkles (Bleszynski 1965). The female of this species was described and figured adequately by Chen et al. (2001).

## Discussion

At present, the genus *Chrysoteuchia* includes 36 species worldwide, and all of them occur in China except for *C.topiaria* (Zeller, 1866) and *C.argentistriellus* (Leech, 1889), which are endemic to North America and Korea, respectively. Among them, 22 species were originally described from 1758 to 1965 (Bleszynski 1965). No species were described between 1965 and 2001, but a renewed interest in the genus added another eleven species in the early 2000’s, all described from China (Chen et al. 2001, 2003; Li and Li 2010; Li and Liu 2012).

In this study, we show that Bio18 (precipitation of the warmest quarter) is the most important variable with respect to the distribution patterns of the genus (Table 1), with most known presence sites located within the regions with 310–867 mm precipitation of the warmest quarter (Fig. 2). However, the region to the south of 24°N, which has suitable precipitation has low logistic values of potential habitats and few recorded localities (Figs 1, 2). This can be explained by the aid of Bio11 (mean temperature of the coldest quarter), the strongest predictor of the temperature variables. According to the response curve of *Chrysoteuchia* to Bio11 (Suppl. material 2: Fig. S1), we can conclude that the suitable temperatures for the *Chrysoteuchia* occurrences are between -40 °C and 25 °C. These manifest that the species of this genus are humidity dependent and cold tolerant but find it difficult to colonise the relatively hot areas. The spectrum of tolerated temperatures in *Chrysoteuchia* suggests a dispersion to higher altitudes or latitudes in some species to avoid the hot weather in South China. Furthermore, some members of the genus may be considered as potential bioindicators with respect to global warming.

### Acknowledgements

Cordial thanks are extended to Dr Jurate De Prins for her kind support while Weichun Li studied the insect collection of the Natural History Museum, London. Special thanks are given to Dr Matthias Nuss, Dr Graziano Bassi, and Théo Léger for their insightful comments and suggestions on the manuscript. This project was supported by the National Natural Science Foundation of China (No. 31601885).

## Supplementary Material

XML Treatment for Chrysoteuchia landryi

XML Treatment for Chrysoteuchia curvicavus

## Supplementary materials

Supplementary material 1 Table S1 Data type: species data Explanation note: Overview of Chinese localities where *Chrysoteuchia* species have been collected (administrative divisions are given in bold) with geographical coordinates in the decimal system. This dataset is made available under the Open Database License (http://opendatacommons.org/licenses/odbl/1.0/). The Open Database License (ODbL) is a license agreement intended to allow users to freely share, modify, and use this Dataset while maintaining this same freedom for others, provided that the original source and author(s) are credited.Lu-Lan Jie, Jing-Bo Yang, Wei-Chun Li

Supplementary material 2 Figure S1 Data type: image Explanation note: Response curve of *Chrysoteuchia* to Bio11 (mean temperature of the coldest quarter). This dataset is made available under the Open Database License (http://opendatacommons.org/licenses/odbl/1.0/). The Open Database License (ODbL) is a license agreement intended to allow users to freely share, modify, and use this Dataset while maintaining this same freedom for others, provided that the original source and author(s) are credited.Lu-Lan Jie, Jing-Bo Yang, Wei-Chun Li

## References

[B1] Bleszynski S (1965) Crambinae. In: Amsel HG Reisser H Gregor F (Eds) Microlepidoptera Palaearctica 1(1–2).Verlag Georg Fromme and Co, Wien, 1–553.

[B2] Chen TM Song SM Yuan DC (2001) The genus *Chrysoteuchia* Hübner (Lepidoptera: Pyralidae: Crambinae) from China with descriptions of five new species. Oriental Insects 35: 177–192. 10.1080/00305316.2001.10417298

[B3] Chen TM Song SM Yuan DC (2003) Two new species of Crambinae (Lepidoptera: Pyralidae: Crambinae). Acta Zootaxonomica sinica 28(3): 521–524.

[B4] Hijmans RJ Cameron SE Parra JL Jones PG Jarvis A Richardson K (2004) Worldclim 1.3. http://www.worldclim.org [accessed 16 December 2018]

[B5] Hübner J (1825) Verzeichnis bekannter Schmettlinge. Augsburg, 432 pp. [1816–1826] 10.5962/bhl.title.48607

[B6] Inoue H (1989) Redescriptions of three species of the Crambinae described by Marumo (1936) from Taiwan (Lepidoptera: Pyralidae). Tinea 12(20): 185–189.

[B7] Landry JF Landry B (1994) A technique for setting and mounting Microlepidoptera. Journal of the Lepidopterists’ Society 48(3): 205–227.

[B8] Landry B (1995) A phylogenetic analysis of the major lineages of the Crambinae and of the genera of Crambini of North America (Lepidoptera: Pyralidae). Memoirs on Entomology International 1: 1–242.

[B9] Li WC Li HH (2010) Four new species of the genus *Chrysoteuchia* Hübner (Lepidoptera: Crambidae: Crambinae) from China. Zootaxa 2458: 33–46. 10.11646/zootaxa.2485.1.3

[B10] Li WC Liu D (2012) *Chrysoteuchianingensis* sp. n., a new pyralid moth (Lepidoptera: Crambidae) from China. Entomologica Fennica 23: 102–106.

[B11] Li WC Liu D (2015) A new species of *Metaeuchromius* (Lepidoptera, Crambidae) from the Tibetan glacier area of China. ZooKeys 475: 113–118. 10.3897/zookeys.475.8766 PMC4311702 25685000

[B12] Li WC Jie LL Liu D (2016) The genus *Scoparia* (Lepidoptera, Crambidae) from Galongla Snow Mountain of China, with DNA barcoding and descriptions of three new species. Systematics and Biodiversity 14(3): 303–313. 10.1080/14772000.2016.1140246

[B13] Li WC (2017) Morphology and molecules reveal high species diversity of *Ligidium* (Crustacea: Oniscidea: Ligiidae) from Jiangxi, China. Zoological Journal of the Linnean Society 179(3): 627–641. 10.1111/zoj.12464

[B14] Li WC (2018) Notes on *Glaucocharis* (Lepidoptera, Crambidae) from China, with descriptions of two new species. Zookeys 807: 149–158. 10.3897/zookeys.807.29237 PMC6305352 30595655

[B15] Li WC (2019) Integrative taxonomy reveals the exceptional species diversity of *Eudonia* (Lepidoptera: Crambidae) in Tibet, China. Systematics and Biodiversity 17(1): 39–50. 10.1080/14772000.2018.1523812

[B16] Phillips SJ Anderson RP Schapire RE (2006) Maximum entropy modeling of species geographic distributions. Ecological Modelling 190(3–4): 231–259. 10.1016/j.ecolmodel.2005.03.026

