# Supplementary material for: The geographical distribution patterns of Chrysoteuchia Hübner in China and description of a new species (Lepidoptera, Crambidae)
Source: Zookeys. 2019 Jun 6;853:109–18. doi: 10.3897/zookeys.853.34149 (PMC6580841; doi:10.3897/zookeys.853.34149)
Supplement: Supplementary material 1 [file zookeys-853-109-s001.docx]

**The geographical distribution patterns of *Chrysoteuchia* Hübner in China and description of a new species (Lepidoptera, Crambidae)**

**Supporting Information**

**Table S1.** Overview of Chinese localities where *Chrysoteuchia* species have been collected (administrative divisions are given in bold) with geographical coordinates in the decimal system

| **Taxa** | **Collection locality** | **Eastern Longitude** | **Northern Latitude** |
| --- | --- | --- | --- |
| *C. atrosignata* | **Anhui**, Huangshan (Li 2010) | 118.3000 | 29.7167 |
|  | Huoshan (Li 2010) | 116.3167 | 31.4000 |
|  | Jiuhuashan (Li 2010) | 117.8000 | 30.3833 |
|  | **Fujian**, Leigongshan, 1600 m | 108.2833 | 26.4667 |
|  | Putian, Xianyou, alt. 780 m (JXAUM) | 118.7000 | 25.3700 |
|  | Wuyishan, alt. 740 m (Li 2010) | 116.7000 | 26.9000 |
|  | **Gansu**, Wenxian, alt. 718–860 m (Li 2010) | 104.6833 | 32.9667 |
|  | **Guangxi**, Jinxiu, alt. 550 m (Li 2010) | 110.1833 | 24.1333 |
|  | Maoershan, alt. 550–1100 m (Li 2010) | 110.4167 | 25.8833 |
|  | Rongshui, alt. 579–650 m (Li 2010) | 109.2167 | 25.0667 |
|  | Yuanbaoshan, alt. 500–700 m (Li 2010) | 109.1667 | 25.3833 |
|  | **Guizhou**, Chishui, alt. 240–390 m (Li 2010) | 105.7000 | 28.5667 |
|  | Daozhen, alt. 600 m (Li 2010) | 107.6000 | 28.8833 |
|  | Fanjingshan, alt. 530–1390 m (Li 2010) | 108.6833 | 27.9167 |
|  | Jiangkou, alt. 600 m (Li 2010) | 108.8333 | 27.6833 |
|  | Leishan, alt. 900 m (Li 2010) | 108.0500 | 26.3667 |
|  | Mayanghe, alt. 700–800 m (Li 2010) | 108.5000 | 28.5500 |
|  | Rongjiang, alt. 680 m (Li 2010) | 108.5000 | 25.9333 |
|  | Xishui, alt. 500 m (Li 2010) | 106.2000 | 28.3167 |
|  | **Hebei**, Weixian, Xiaowutai, alt. 800 m (Li 2010) | 115.0333 | 39.9500 |
|  | **Henan**, Gushi (Li 2010) | 115.6833 | 32.1667 |
|  | Luoshan, alt. 350 m (Li 2010) | 114.5167 | 32.2000 |
|  | Lushi, alt. 1200 m (Li 2010) | 111.0333 | 34.0500 |
|  | Neixiang, alt. 1350 m (Li 2010) | 111.0833 | 33.0333 |
|  | Tongbai, alt. 300 m (Li 2010) | 113.4000 | 32.3500 |
|  | Xinyang, jigongshan, alt. 1200 m (Li 2010) | 114.0667 | 32.1667 |
|  | Xixia, alt. 890 m (Li 2010) | 111.4833 | 33.3000 |
|  | **Hubei**, Hefeng, alt. 1200 m (Li 2010) | 110.0333 | 29.8833 |
|  | Lichuan, alt. 700 m (Li 2010) | 108.9333 | 30.3000 |
|  | Xianfeng, alt. 400 m (Li 2010) | 109.1333 | 29.6667 |
|  | **Hunan**, Shimen, Hupingshan, 1200 m (JXAUM) | 111.3667 | 29.5833 |
|  | Taojiang (Li 2010) | 112.1333 | 28.5333 |
|  | Xiangtan (Li 2010) | 112.8833 | 27.8667 |
|  | Xinhua (Li 2010) | 111.3000 | 27.7333 |
|  | **Jiangxi**, Dexing (Li 2010) | 117.5833 | 28.9500 |
|  | Ganzhou, Dingnan, Yuntaishan, alt. 763 m (JXAUM) | 115.0300 | 24.7800 |
|  | Ganzhou, Fengshan (Li 2010) | 114.9333 | 25.8667 |
|  | Ganzhou, Jiulianshan (Chen et al. 2001) | 114.5500 | 24.6333 |
|  | Ganzhou, Xunwu, Xiangshan (Li 2010) | 115.6333 | 24.9500 |
|  | Jingdezheng, Fuliang, alt. 220 m (JXAUM) | 117.2500 | 29.3700 |
|  | Jiujiang, Wuning (JXAUM) | 115.1667 | 29.4500 |
|  | Nanchang, Agricultural University, alt. 50 m (JXAUM) | 115.9500 | 28.6800 |
|  | Nanchang, Jinxian (JXAUM) | 116.2700 | 28.3700 |
|  | Pingxiang, Matian, alt. 250–350 m (JXAUM) | 114.0300 | 27.6300 |
|  | Shangrao, Geyang (Li 2010) | 117.9667 | 28.4500 |
|  | Shangrao, Guangfeng, alt. 400–900 m (JXAUM) | 118.1800 | 28.4300 |
|  | Shangrao, Yanshan, Wuyishan, 900–1820 m (JXAUM) | 117.7000 | 28.3200 |
|  | Shangrao, Yushan, Sanqingshan, alt. 380–450 m (JXAUM) | 117.9667 | 28.4500 |
|  | Yichun, Jing'an, Sanzhualun, alt. 220 m (JXAUM) | 115.3500 | 28.8700 |
|  | Yichun, Tonggu, alt. 800 m (JXAUM) | 114.3700 | 28.5300 |
|  | Yichun, Yifeng, Guanshan, alt. 160–400 m (JXAUM) | 114.7800 | 28.3800 |
|  | **Shaanxi**, Xixiang, alt. 760 m (Li 2010) | 107.7667 | 32.1500 |
|  | Yangxian, alt. 420–680 m (Li 2010) | 107.5500 | 33.2167 |
|  | Yangling, alt. 450 m (Li 2010) | 108.0667 | 34.2833 |
|  | **Shanxi**, Baoxing, alt. 1600 m (Li 2010) | 102.8333 | 35.5000 |
|  | Jicheng, Lishan, alt. 1520 m (Li 2010) | 112.8500 | 35.5000 |
|  | **Shandong**, Yantai, Kunyushan (Li 2010) | 121.6667 | 37.2000 |
|  | **Shanghai** (Chen et al. 2001) | 121.4833 | 31.2333 |
|  | **Sichuan**, Emeishan (Chen et al. 2001) | 103.3167 | 29.5333 |
|  | Luxian (Li 2010) | 105.4333 | 28.9333 |
|  | Jianyang, alt. 350–400 m (Li 2010) | 104.5333 | 30.4000 |
|  | Mabian, alt. 1100 m | 103.5167 | 28.8500 |
|  | Wolong, alt. 2008 m (Li 2010) | 103.6000 | 31.4833 |
|  | **Yunnan**, Dali, Hudiequan, alt. 2030 m (Li 2010) | 100.2167 | 25.5667 |
|  | Lijiang, Shizishan, alt. 2400 m (Li 2010) | 100.2333 | 26.8667 |
|  | Longling, Xiaoheishan, alt. 2300 m (Li 2010) | 98.6833 | 24.5833 |
|  | **Zhejiang**, Lin’an, Shunxi, alt. 420 m (Li 2010) | 118.9333 | 30.0667 |
|  | Lishui, Fengyangshan, alt. 1470 m (Li 2010) | 119.9000 | 28.4000 |
|  | Taishun, Wuyanling, alt. 400–930 m (Li 2010) | 119.7000 | 27.5500 |
|  | Tianmushan, alt. 350–800 m (Li 2010) | 119.5667 | 30.4333 |
| *C. culmella* | **Heilongjiang**, Tahe (Li 2010) | 124.7333 | 52.3167 |
|  | **Xingjiang**, Yili (Chen et al. 2001) | 81.3333 | 43.9167 |
| *C. curvicavus* | **Fujian**, Wuyishan, alt. 1800 m (Chen et al. 2001) | 116.7000 | 26.9000 |
|  | **Sichuan**, Mabian, alt. 1100 m | 103.5167 | 28.8500 |
| *C. daisetsuzana* | **Yunnan**, Lijiang (Chen et al. 2001) | 100.2333 | 26.8667 |
| *C. deltella* | **Heilongjiang**, Dailing, alt. 390 m (Chen et al. 2001) | 129.0333 | 47.0333 |
| *C. dentatella* | **Fujian**, Wuyishan, alt. 740 m (Li 2010) | 116.7000 | 26.9000 |
|  | **Gansu**, Tianshui, alt. 1331–1342 m (Li 2010) | 105.7000 | 34.6167 |
|  | Wenxian, Qiujiaba, alt. 2350 m (Chen et al. 2001) | 104.6833 | 32.9667 |
|  | **Shaanxi**, Ningshan, alt. 1360 m | 108.3333 | 33.3167 |
| *C. diplogramma* | **Fujian**, Wuyishan, alt. 740 m (Li 2010) | 116.7000 | 26.9000 |
|  | **Heilongjiang**, Dailing (Chen et al. 2001) | 129.0333 | 47.0333 |
|  | Liangshui National Nature Reserves (Li 2010) | 129.0333 | 47.0333 |
|  | Maoershan (Li 2010) | 127.5000 | 45.2667 |
|  | **Liaoning**, Kuandian (Li 2010) | 124.7667 | 40.7167 |
|  | **Sichuan**, Wanxian (Chen et al. 2001) | 108.3500 | 30.8333 |
|  | **Zhejiang**, Tianmushan (Caradja & Meyrick 1935) | 119.5667 | 30.4333 |
| *C. disasterella* | **Hebei**, Laiyuan, Baishishan, alt. 1300 m (Li 2010) | 114.6833 | 39.2000 |
|  | Jinxing, Xiantaishan, alt. 1200 m (Li 2010) | 114.0167 | 38.0167 |
|  | Neiqiu, alt. 670 m (Li 2010) | 114.5000 | 37.3000 |
|  | Xinglong, Wulingshan (Li 2010) | 117.4833 | 40.6000 |
|  | **Henan**, Huixian, alt. 780 m (Li 2010) | 111.0333 | 34.0500 |
|  | Lushi, alt. 1200–1600 m (Li 2010) | 111.0333 | 34.0500 |
|  | Neixiang, alt. 1200 m (Li 2010) | 111.0833 | 33.0333 |
|  | Shanxian, Ganshan, alt. 1100 m (Li 2010) | 111.2000 | 34.4500 |
|  | **Hubei**, Xingshan (Chen et al. 2001) | 110.7333 | 31.2167 |
|  | **Ningxia**, Liupanshan, alt. 1700 m (Li 2010) | 106.1833 | 35.7000 |
|  | **Shaanxi**, Ankang, Hualongshan, alt. 2150 m (Li 2010) | 109.0167 | 32.6833 |
|  | **Tianjin**, Jizhou, alt. 300 m (Li 2010) | 117.4000 | 40.0333 |
| *C. distinctella* | **Fujian**, Wuyishan (Chen 2003) | 116.7000 | 26.9000 |
|  | **Heilongjiang**, Dailing (Chen et al. 2001) | 129.0333 | 47.0333 |
|  | Maoershan (Li 2010) | 127.5000 | 45.2667 |
|  | Yichun (Chen et al. 2001) | 128.9333 | 47.7000 |
| *C. dividella* | **Tibet**, Cuona (Chen et al. 2001) | 91.9333 | 27.9667 |
|  | Jilong (Chen et al. 2001) | 85.3500 | 28.3833 |
|  | Quxiang (Chen et al. 2001) | 85.9833 | 28.0833 |
| *C. fractella* | **Sichuan**, Emeishan (Bleszynski 1965) | 103.3167 | 29.5333 |
| *C. fuliginosella* | **Sichuan**, Gonggashan (Chen et al. 2001) | 101.8667 | 29.5833 |
| *C. funebrella* | **Yunnan**, Lijiang (Bleszynski 1965) | 100.2333 | 26.8667 |
| *C. furva* | **Ningxia**, Liupanshan (Li & Li 2010) | 106.1833 | 35.7000 |
|  | **Gansu**, Kangxian (Li & Li 2010) | 105.6000 | 33.3333 |
| *C. gonoxes* | **Yunnan**, Dali, Diancangshan, alt. 2900 m (Li 2010) | 100.2167 | 25.5667 |
|  | Hushui (Chen et al. 2001) | 98.8667 | 25.8667 |
|  | Lijiang (Chen et al. 2001) | 100.2333 | 26.8667 |
| *C. gregorella* | **Heilongjiang**, Dailing (Chen et al. 2001) | 129.0333 | 47.0333 |
|  | **Jilin**, Changbaishan (Chen et al. 2001) | 128.1333 | 42.0333 |
|  | **Sichuan**, Wanxian (Chen et al. 2001) | 108.3500 | 30.8333 |
| *C. hamatella* | **Tibet**, Yadong, alt. 4500 m (Chen et al. 2001) | 88.9667 | 27.5167 |
| *C. hamatoides* | **Qinghai**, Yushu, alt. 3980 m (Chen et al. 2001) | 96.9833 | 33.0333 |
|  | **Shaanxi**, Ningshan (Li 2010) | 108.3333 | 33.3167 |
| *C. hyalodiscella* | **Shaanxi**, Ningshan (Li 2010) | 108.3333 | 33.3167 |
|  | **Sichuan**, Kangding [Tatsienlu] (Bleszynski 1965) | 101.9500 | 30.0667 |
|  | Songpan [Sunpanting] (Bleszynski 1965) | 103.5833 | 32.6500 |
| *C.landryi* | **Tibet**, Mêdog, Galongla Snow Mountain, alt. 3415 m (JXAUM) | 95.6768 | 29.7382 |
| *C. lolotiella* | **Sichuan**, Kangding [Tatsienlu] (Bleszynski 1965) | 101.9500 | 30.0667 |
|  | Songpan [Sunpanting] (Bleszynski 1965) | 103.5833 | 32.6500 |
| *C. mandschurica* | **Heilongjiang**, Dailing (Chen et al. 2001) | 129.0333 | 47.0333 |
|  | **Hubei**, Wuchang (Chen et al. 2001) | 114.2833 | 30.5333 |
| *C. moriokensis* | **Fujian**, Wuyishan (Chen 2003) | 116.7000 | 26.9000 |
| *C. ningensis* | **Ningxia**, Longde (Li & Liu, 2012) | 106.1167 | 35.6167 |
| *C. nonifasciaria* | **Tibet**, Mêdog, Galongla Snow Mountain, alt. 3415 m (JXAUM) | 95.6768 | 29.7382 |
|  | Mêdog, Laka (Li & Li 2010) | 95.6000 | 29.2167 |
| *C. picturatella* | **Sichuan**, Emeishan (Chen et al. 2001) | 103.3167 | 29.5333 |
|  | Washan (Bleszynski 1965) | 103.3667 | 29.9333 |
| *C. porcelanella* | **Fujian**, Wuyishan (Chen 2003) | 116.7000 | 26.9000 |
|  | **Heilongjiang**, Dailing (Chen et al. 2001) | 129.0333 | 47.0333 |
|  | Liangshui National Nature Reserves (Li 2010) | 129.0333 | 47.0333 |
|  | **Jilin**, Erdao, alt. 760–1010 m (Li 2010) | 125.6500 | 43.3500 |
|  | **Liaoning**, Huairen (Li 2010) | 125.3500 | 41.2500 |
|  | Kuandian, Shilazi National Nature Reserves (Li 2010) | 124.7667 | 40.7167 |
| *C. pseudodiplogramma* | **Yunnan**, Zhenxiong (Chen et al. 2001) | 104.8667 | 27.4500 |
| *C. quadrapicula* | **Guizhou**, Daozhen, alt. 1300–1370 m (Li 2010) | 107.6000 | 28.8833 |
|  | Fanjingshan, alt. 1300 m (Li 2010) | 108.6833 | 27.9167 |
|  | **Henan**, Luoyang, Baiyunshan, alt. 1560 m (Li 2010) | 112.4500 | 34.6833 |
|  | Neixiang, alt. 1350 m (Li 2010) | 111.0833 | 33.0333 |
|  | Xixia, alt. 890 m (Li 2010) | 111.4833 | 33.3000 |
|  | Yiyang, Huaguoshan, alt. 1000 m (Li 2010) | 112.1667 | 34.5167 |
|  | **Hubei**, Hefeng, alt. 1260 m (Li 2010) | 110.0333 | 29.8833 |
|  | Shengnongjia, alt. 1100 m (Li 2010) | 110.6667 | 31.7500 |
|  | Xianfeng, alt. 1260 m (Li 2010) | 109.1333 | 29.6667 |
|  | **Hunan**, Sangzhi, Badagongshan, alt. 1250 m (Li 2010) | 110.1833 | 29.3833 |
|  | **Zhejiang**, Lishui, Fengyangshan, alt. 1444–1470 m (JXAUM) | 119.9000 | 28.4000 |
| *C. pyraustoides* | **Heilongjiang**, Dailing (Chen et al. 2001) | 129.0333 | 47.0333 |
|  | Mudanjiang (Chen et al. 2001) | 129.6000 | 44.5833 |
|  | Yichun (Chen et al. 2001) | 128.9333 | 47.7000 |
|  | **Ningxia**, Liupanshan (Chen et al. 2001) | 106.1833 | 35.7000 |
|  | **Shaanxi**, Yangling (Chen et al. 2001) | 108.0667 | 34.2833 |
|  | **Tibet**, Chaya (Chen et al. 2001) | 97.5333 | 30.6833 |
| *C. rotundiprojecta* | **Gansu**, Tianshui, alt. 1342 m (Li & Li 2010) | 105.7000 | 34.6167 |
| *C. shafferi* | **Shaanxi**, Ningshan, alt. 2100–2400 m (Li & Li 2010) | 108.3333 | 33.3167 |
| *C. sperlingi* | **Tibet**, Mêdog, Galongla Snow Mountain, alt. 3415 m (JXAUM) | 95.6768 | 29.7382 |
| *C. sonobei* | **Taiwan** (Yoshiyasu 1992) | 121.5000 | 25.0500 |
| *C. yuennanella* | **Yunnan**, Lijiang (Bleszynski 1965) | 100.2333 | 26.8667 |


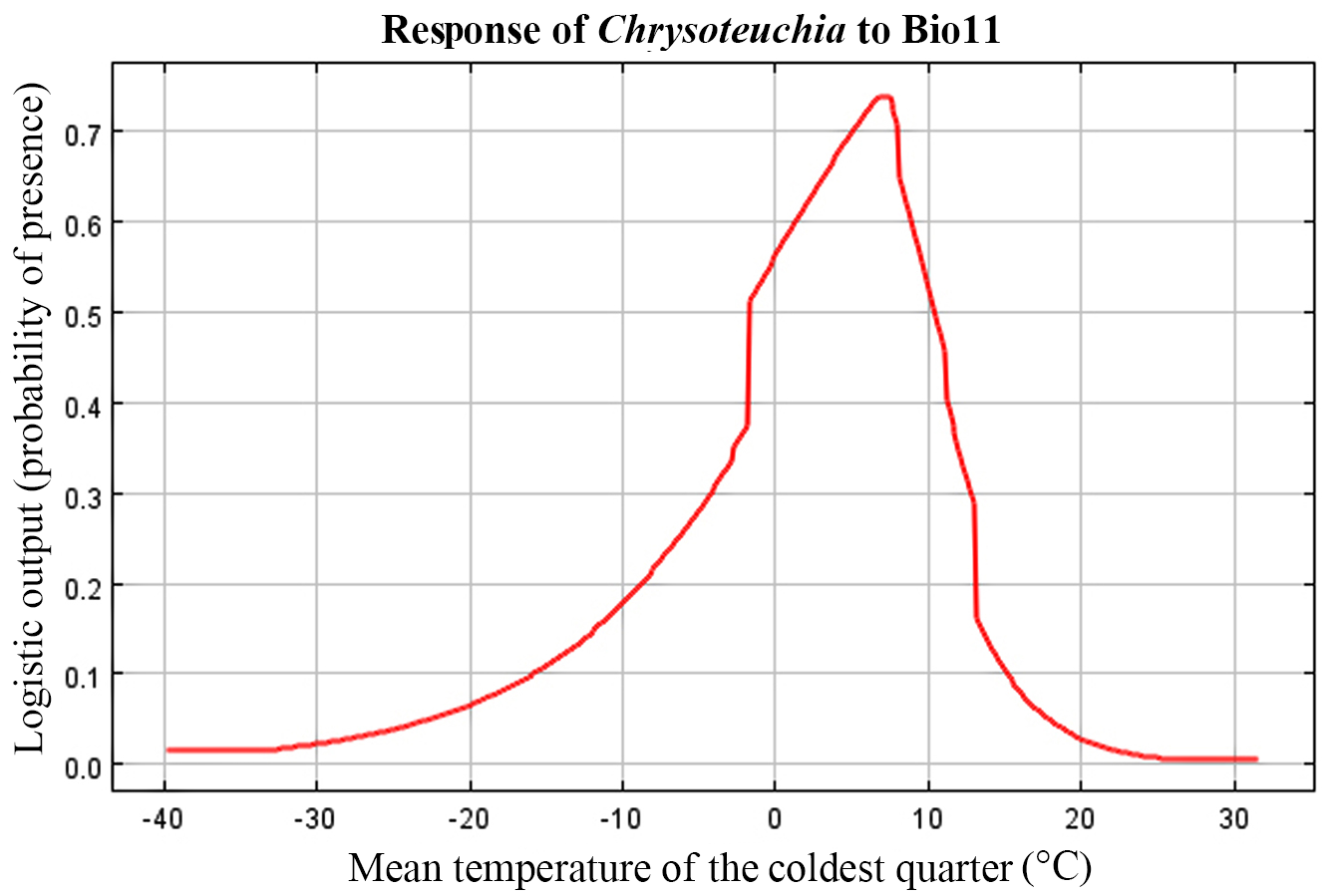


**Figure S1.** Response curve of *Chrysoteuchia* to Bio11 (mean temperature of the coldest quarter)
