# Supplementary figures and images for: The geographical distribution patterns of Chrysoteuchia Hübner in China and description of a new species (Lepidoptera, Crambidae)
Source: Zookeys. 2019 Jun 6;853:109–18. doi: 10.3897/zookeys.853.34149 (PMC6580841; doi:10.3897/zookeys.853.34149)

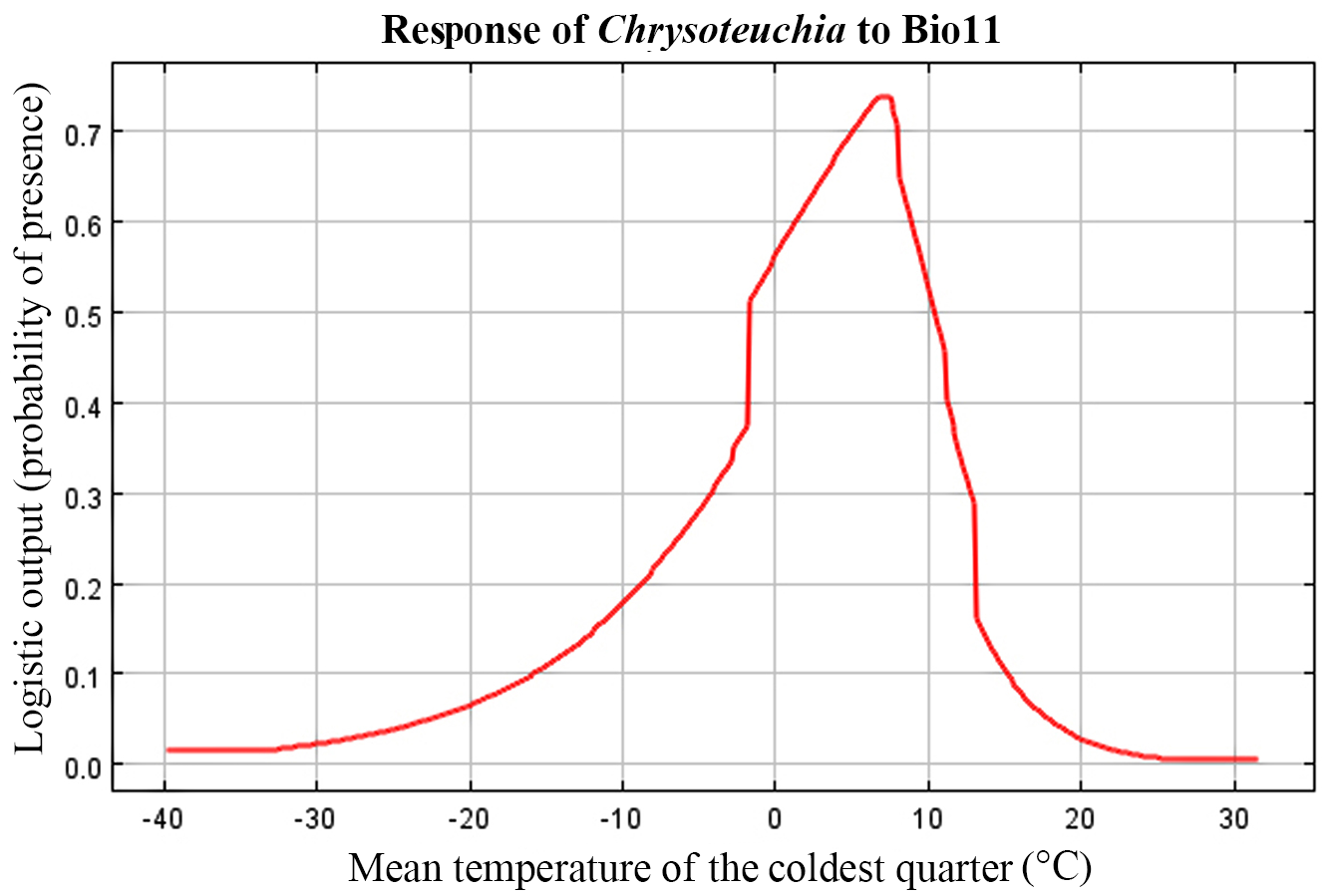

Supplement: Supplementary material 2 [file zookeys-853-109-s002.tif]
